# A comprehensive Bayesian analysis assessing the effectiveness of lymphocyte immunotherapy for recurrent spontaneous abortion

**DOI:** 10.1093/lifemedi/lnad049

**Published:** 2023-12-08

**Authors:** Rongzhou Chen, Haohan Xu, Yujia Hou, Hanghang Liu, Zheng Zheng, Shaohua Ma

**Affiliations:** Tsinghua Shenzhen International Graduate, Tsinghua University, Shenzhen 518055, China; Tsinghua-Berkeley Shenzhen Institute, Shenzhen 518055, China; Tsinghua Shenzhen International Graduate, Tsinghua University, Shenzhen 518055, China; Tsinghua Shenzhen International Graduate, Tsinghua University, Shenzhen 518055, China; Tsinghua-Berkeley Shenzhen Institute, Shenzhen 518055, China; Tsinghua Shenzhen International Graduate, Tsinghua University, Shenzhen 518055, China; Shenzhen Maternity and Child Healthcare Hospital, Shenzhen 518028, China; Tsinghua Shenzhen International Graduate, Tsinghua University, Shenzhen 518055, China; Tsinghua-Berkeley Shenzhen Institute, Shenzhen 518055, China

**Keywords:** recurrent spontaneous abortion, lymphocyte immunotherapy, Bayesian analysis, Bayesian meta-analysis

## Abstract

Recurrent spontaneous abortion (RSA) affects 2%–5% of couples worldwide and remains a subject of debate regarding the effectiveness of lymphocyte immunotherapy (LIT) due to limited retrospective studies. We conducted a comprehensive Bayesian analysis to assess the impact of LIT on RSA. Using data from the Shenzhen Maternity and Child Healthcare Hospital (2001–2020, *n* = 2316), a Bayesian generalized linear model with predictive projection feature selection was employed. Our analysis revealed a significant improvement in live birth rates for RSA patients undergoing LIT. Notably, LIT had a greater impact compared to the other 85 factors considered. To mitigate research bias, we conducted a Bayesian meta-analysis combining our dataset with 19 previously reported studies (1985–2021, *n* = 4246). Additionally, we developed an empirical model highlighting the four key factors, which are the LIT result, age, paternal blood type, and anticardiolipin antibody. Younger age (19–27), paternal blood type B, and a positive anticardiolipin antibody (IgM) were associated with better therapeutic outcomes in LIT for RSA. These findings aid clinicians in identifying suitable candidates for LIT and improving treatment outcomes.

## Introduction

Recurrent spontaneous abortion (RSA), defined as experiencing two or more miscarriages, is a widespread issue affecting millions of couples worldwide [1, 2]. Globally, an estimated 2%–5% of couples are suffering from RSA [3]. Numerous studies have demonstrated that immune factors account for over 60% of unexplained RSA, especially for alloimmune disorders (a lack of maternal blocking antibodies (BAs)), resulting in the maternal immune system attacking the embryo or foetus [4]. Therefore, immune therapies are considered to be the most effective treatment for RSA.

Lymphocyte immunotherapy (LIT) has been widely used to treat RSA patients who test negative for BAs [5] in an attempt to increase their BAs until the patient’s test comes positive. LIT was initially shown effective and was practiced in clinics in the United States from 1985 [6] until the FDA prohibited its use in 2002 when clinical studies showed no significant increase in live births on RSA patients [7]. In China, LIT has been in use since 1987, when increased birth rates in RSA patients were reported [8, 9]. However, the safety and efficacy of LIT remain controversial. For instance, five patients were infected with HIV in Zhejiang in 2017 [10], resulting in an irreversible medical incident. Due to this, LIT should be implemented when its benefits outperform risks. Therefore, there is an urgent need to evaluate the effectiveness and risks of LIT, and, should this therapy prove effective, the analysis of individual influential factors on treatment outcomes could assist clinicians in selecting the most appropriate patient candidates for LIT.

Several factors may influence the effectiveness of LIT, including patients’ medical history, infections, endocrine disorders [11], immune factors, thrombophilia, and chromosomal disorders [12]. In this study, we examined 86 basic conditions of the patient and identified them as factors that may affect delivery, encompassing patients’ medical history, infection, prothrombotic state, as well as endocrine, immune, chromosomal, and paternal factors. Since LIT makes use of allogeneic immune cells, the physical condition of the male partner may influence its efficacy [13]. Therefore, data from some male routine examinations including semen and blood analyses were included in our study. For more details on the selection of influential factors, please refer to the ‘Data availability’ section. In this paper, we constructed a Bayesian generalized linear model (Bayesian GLM) to assess the outcomes of LIT, considering the various conditions of the patients. The results of our study demonstrated that the influence of LIT on the live birth rate was the predominant factor. Then to avoid potential biases that may have resulted in the findings of the Bayesian GLM, we conducted a Bayesian meta-analysis, which yielded the same conclusion as the Bayesian GLM. Finally, in order to identify the best candidates for LIT efficiently and practically, we modelled an empirical model. We demonstrated the effectiveness of LIT in treating RSA and our work can assist clinical doctors in identifying the most suitable patients for LIT more effectively.

## Results

### Bayesian generalized linear model

To establish the relationship between childbirth and the patient’s basic physical condition, we initially employed a Bayesian GLM [14]. The data utilized for this analysis were obtained from the Shenzhen Maternity and Child Healthcare Hospital, covering the period from 2001 to 2020. We considered 86 basic conditions that were examined as potential factors influencing the delivery outcome. The success of giving birth was taken as the indicator, making it a logistic regression problem. The model was formulated as follows:


$$\begin{array}{l} \ln\left(\frac{p(y=1|X)}{1-p(y=1|X))}\right)∼\beta_{0}+\beta_{1}x_{1}+\beta_{2}x_{2}+\cdots +\beta_{86}x_{86}, \end{array}$$

Here, X represents the vector of 86 factors ($x_{1},\;x_{2},\;\ldots ,x_{86}$) and p(y=1|X) denotes the probability of successfully giving birth predicted by the model for a given set of conditions X. In other words, it represents the predicted probability of a patient successfully giving birth to a child based on the specific combination of factors $\left(x_{1},\;x_{2},\;\ldots ,x_{86}\right)$ where $x_{i}$ (i=1, 2, 3,…, 86) corresponds to various factors that may contribute to RSA.

A Bayesian perspective for Modelling a hierarchical GLM provides several advantages over alternative theoretical methods used to approximate complex likelihood functions, typically employed in the frequentist framework [15, 16]. Bayesian analysis accounts for the uncertainty in the estimation of prior distribution characteristics of model parameters, making the results more reliable [17, 18]:

Firstly, Bayesian analysis allows for the incorporation of prior knowledge or clinician opinions in the form of prior distributions. This integration of existing information or expertise helps to improve the estimation process, leading to more accurate and reliable results. This allows us to fully utilize the large amount of prior knowledge that leads to RSA that we have collected, detailed prior settings can be found in Supplementary Table S4.

Secondly, Bayesian analysis proves particularly valuable when dealing with small sample sizes, which is often the case in medical studies. With limited data, frequentist methods may yield unstable or unreliable estimates. However, Bayesian analysis addresses this issue by leveraging prior knowledge, stabilizing the estimates, and providing more dependable outcomes. By incorporating prior information, the impact of data scarcity is reduced, allowing for more accurate predictions and robust conclusions.

We conducted a thorough evaluation of our Bayesian GLM to ensure its accuracy before analysing the impact of various factors on the indicator [19]. The model exhibited no divergence, and values of $\hat{R}$ were all close to 1 [20]. The effect sample size (ESS) of the parameters was sufficiently large, indicating accurate parameter estimations [21]. The specific values of $\hat{R}$ and ESS are exhibited in Supplementary material 1. Moreover, the Bayesian $\;R^{2}$ exceeds 0.3, further supporting the goodness of fit of our mode (see Supplementary material 2). We also performed a sample posterior predictive check on Bayesian GLM (Supplementary Fig. S1A) and empirical model (Supplementary Fig. S1B), which demonstrated no significant difference between the observed and predicted data. This finding indicates that our model exhibited good fitting ability [21]. Furthermore, all four Markov chains of Bayesian GLM (Supplementary Fig. S2A), empirical model (Supplementary Fig. S2B), and Bayesian meta-analysis model (Supplementary Fig. S2C), as determined in our analysis, converged to the same region. This convergence indicates that the chains thoroughly explored the posterior distribution and reached a stable state. Consequently, we can confidently assert that the estimates of the model parameters are reliable [22].

**Figure 1.**
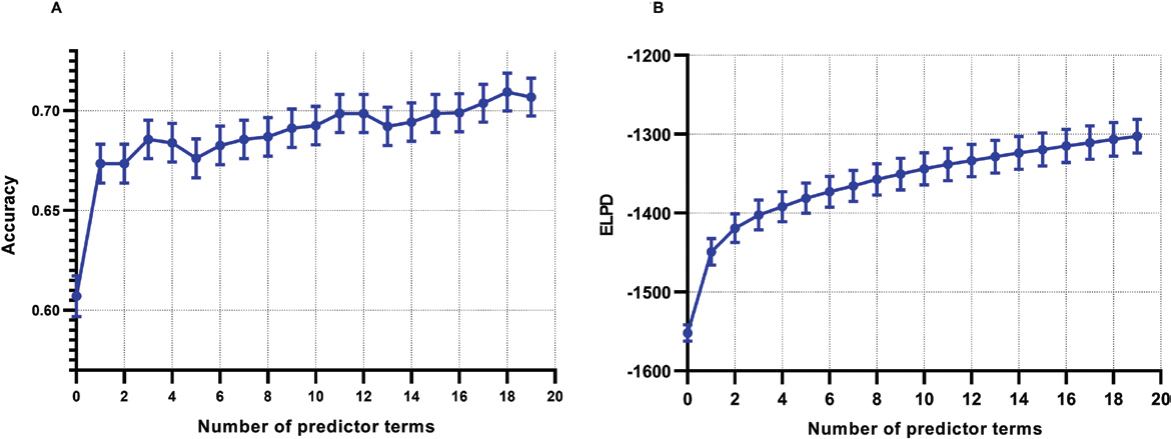
**Analysis of the trend in predictive powers with increasing ranked features.** The (A) accuracy and (B) ELPD of the Bayesian model are determined by 19 features based on Predictive Projection Feature Selection (PPFS), they are: BA treatment result, age, paternal blood type, anticardiolipin antibody IgM, and so on. The error bars represent standard deviation. For more details about selected features, see Supplementary Table S9.

To identify the factors influencing childbirth outcomes, we employed Predictive Projection Feature Selection (PPFS) [23] to rank the factors’ impact on fertility indicators. PPFS is a technique used to identify the most influential factors or variables in predicting a particular outcome. By utilizing PPFS, we were able to assess and rank the significance of various fertility indicators in relation to the outcome of interest. This approach allows us to prioritize and highlight the factors that have the greatest impact on fertility outcomes, providing valuable insights for understanding, and addressing reproductive health issues. The results of FFPS are shown in Fig. 1A and B, we draw the top 19 (projpred package default) most important features identified by our Bayesian GLM model. Notably, our analysis revealed that LIT has a significant impact on reducing RSA. Whether to undergo LIT emerged as the most influential factor affecting RSA outcomes. The predictive performance of LIT, as measured by accuracy and expected log predictive density (ELPD) [24], was found to be nearly as significant as the combined effect of the other 85 factors in our model. These findings provide compelling evidence supporting the effectiveness of LIT as a treatment approach for RSA based on our dataset and analysis methods. The prominent impact of LIT on RSA outcomes further reinforces its importance in addressing reproductive health concerns.

### Bayesian meta-analysis

While our data and methodology provided evidence of the significant influence of LIT on increasing birth rates, it is important to acknowledge that biases in the data could potentially impact the research results. Geographical bias and sampling bias are among the factors that may introduce biases into the study. To ensure that the effectiveness of LIT in the treatment of RSA is not limited to the specific location of data collection and to address potential research biases, we decided to establish a Bayesian hierarchical model for conducting a Bayesian meta-analysis. In the meta-analysis, we combined our dataset with data from 19 previous studies, resulting in a total of 6562 patients (refer to Supplementary Table S8 for details). These 19 previous studies, published or unpublished, were conducted between 1985 and 2021. A comprehensive search strategy was employed to identify relevant studies, and rigorous study selection criteria were applied. More details can be found in the ‘Data collection and processing’ section.

In the Bayesian meta-analysis model, we make the assumption that the observed effect size, denoted as $Y_{J}$, from study J serves as an estimate of the ‘true’ effect, represented by $\theta_{J}$ within that specific study. To present the results clearly, we express $Y_{J}$ as the natural logarithm of the relative risk, given by $Y_{J}=lnp_{1}-lnp_{0}$, where $p_{1}=d_{1}/n_{1}$ and $p_{0}=d_{0}/n_{0}$. Here, $d_{1}$ and $d_{0}$ represent the number of patients who gave birth to a child in the treatment and control groups, respectively, while $n_{1}$ and $n_{0}$ represent the total number of patients in the treatment and control groups, respectively.

In this model, we do not explicitly specify the prior distributions for μ (the overall mean effect size) and $\tau^{2}$ (the heterogeneity or between-study variance). Instead, we assign them uninformed prior distributions, which correspond to unrestricted uniform distributions (following Stan’s default setting). With these uninformed priors, we define the model as follows:


$$\begin{array}{l} Y_{J}∼Normal(\theta_{J},\sigma_{J}^{2}) \end{array}$$

$$\begin{array}{l} \theta_{J}∼Normal(\mu ,\tau^{2}) \end{array}$$

**Figure 2.**
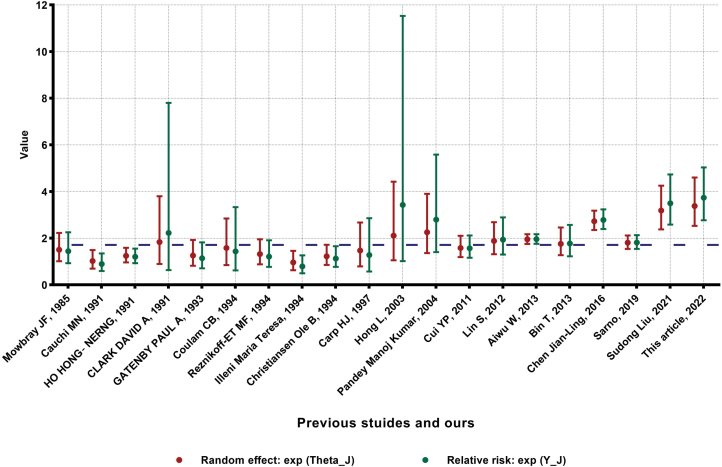
**Result of Bayesian meta-analysis.** The two vertical lines represent the 95% CI of the relative risk $e^{Y_{J}}$ (where $Y_{J}$ is the logarithm of the relative risk) and the random effect $e^{\theta_{J}}$, and the horizontal thick dashed line represents overall mean $e^{\mu },\;$ respectively.

In our Bayesian meta-analysis, $\sigma_{J}$ represents the standard error of the effect estimate in study J, and $\sigma_{J}^{2}$ is assumed to be known with certainty. This assumption is made because, in the case of the binomial distribution and with large sample sizes, each study’s variance can be estimated precisely. The overall mean, denoted as μ, is commonly estimated by taking posterior sampling in Bayesian analysis. This estimate represents the average effect across all included studies. The parameter $\tau^{2}$ reflects the between-study variance. It captures the amount of heterogeneity or variability in effect sizes among the studies. As shown in Fig. 2, a significant shrinkage effect towards the overall mean μ is observed in the estimated effects $\theta_{J}$. This shrinkage effect is more pronounced in studies where the relative risk is estimated less accurately [25]. This indicates that the results obtained through Bayesian approaches are more reliable within each study, as they account for the uncertainty in the estimates and incorporate information from other studies.

In our analysis, we estimated the posterior value of $e^{\mu }$ (the blue dashed line in Fig. 2) to be 1.70 (1.36–2.11; 95% credible interval), indicating that the average effect across all included studies suggests a 1.70-fold increase in the likelihood of successful childbirth for patients undergoing LIT compared to those in the control groups. Additionally, the posterior value of τ was estimated to be 0.42 (0.28–0.64; 95% credible interval), suggesting moderate heterogeneity or variability in effect sizes among the studies. Furthermore, the confidence interval for the random effect $e^{\theta_{J}}$ was 1.70 (0.71–4.13; 95% credible interval), indicating that the effect of LIT on childbirth can vary across different studies. However, the lower bound of the confidence interval suggests a potential decrease in the likelihood of successful childbirth, while the upper bound suggests a substantial increase. This variation highlights the importance of considering individual study effects when interpreting the overall treatment effect.

Based on these findings, we can conclude that LIT is an effective treatment for RSA, as indicated by the higher likelihood of successful childbirth in the treatment groups compared to the control groups. However, it is essential to consider the variability across studies and the wide confidence interval, which may be attributed to differences in study characteristics, patient populations, or other factors.

### Empirical model

In addition to LIT, studies have suggested that there may be other factors such as anti-endometrial [26] or and anti-ovarian antibodies [27] contributing to RSA treatment outcomes. Additionally, Maria *et al.* found that abortion risk increased from 9.8% for 25–29-year-old patients to 33.2% for patients aged 40 and above [28]. Cavalcante *et al.* conducted a meta-analysis that suggested that positive infertility-related antinuclear antibodies [29] might increase the risk of RSA. Moreover, ABO blood group mismatch is the most common cause of alloimmune abnormalities in RSA, that is, the blood type inherited from the father may cause the foetus to be attacked by the mother’s immune system [30]. Additionally, ACAIgM, lupus anticoagulant, and anti-β  2GP1 are considered conventional pathogenic antibodies [31, 32]. Studies have shown that the presence of one or more of these antibodies in women increases the risk of miscarriages, with only a 30% chance of giving birth when all three antibodies are positive [33]. Since these factors can have a significant influence on abortion risk, we decided to investigate their potential interactions with LIT.

**Figure 3.**
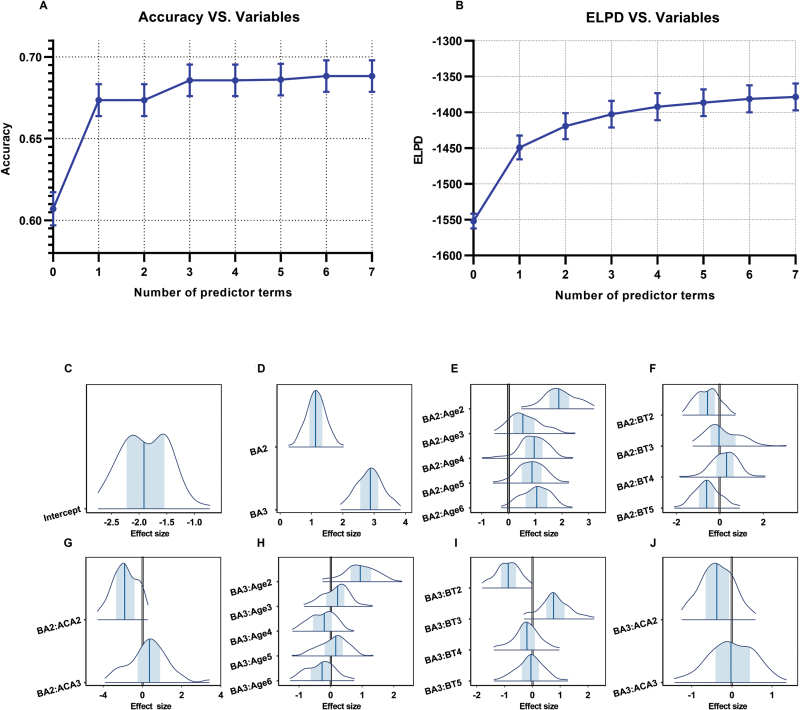
**Predictive performance of the empirical model.** (A) Accuracy and (B) ELPD. The error bars of (A) and (B) represent standard deviation of accuracy and ELPD. (C) Overall intercept of the empirical model. (D) Main effect of LIT outcome, there are significant differences between each level. (E–J) Interaction of LIT with other factors when (E–G) BAs are negative or (H–J) positive. The first level of each factor value is missing value except for LIT outcome (i.e. untreated patients), which was used as a reference level. BA3 and BA2 refer to patients with or without BA conversion, respectively. Age2–Age6 refer to patients aged between 19–27, 27–29, 29–31, 31–34, and 34–57 years old. BT2–BT5 refer to the paternal blood types A, AB, B, or O. ACAIgM2–ACAIgM3 refers to patients with anticardiolipin antibody IgM tested negative or positive, respectively. The vertical lines in panels (C–J) represent the mean effect size for each level within every feature. Additionally, the blue shadows represent the 95% credible intervals of the effect size.

To comprehensively consider the interpretability and predictive performance of all factors, we chose the first four features from our PPFS. These features, namely LIT outcome, age, paternal blood type, and anticardiolipin antibody IgM (ACAIgM), were chosen as they collectively accounted for over 73% of accuracy and 63% of ELPD among the 19 features considered (Fig. 3A and B). An empirical model was established by considering the main effect [34] of overall intercept, LIT outcome, and the interaction of LIT outcomes with age, blood type, and ACAIgM in terms of birth rates (Fig. 3C–J). We also checked the correctness of the empirical model, and it appeared to be valid, as shown in Supplementary materials 3 and 4, values of $\hat{R}$ were all around 1 and the ESS of parameters was sufficiently large. Additionally, the empirical model exhibits a consistent trend in posterior predictive check between predicted and observed data (Supplementary Fig. S1B). Furthermore, the Markov chains have converged to the same region (Supplementary Fig. S2B), indicating it reached a stable state and the estimates of the model parameters are reliable.

Fig. 3E–J clearly illustrates the positive impact of LIT on the live birth rate, regardless of the presence of BAs. Moreover, it demonstrates that the improvement in live birth rate is more significant when BAs are converted to a negative status (BA3) compared to situations where no change in BAs occurs (BA2) or when no treatment is administered (reference level) (Fig. 3D). Furthermore, the interaction analysis reveals that age plays a particularly significant role in the outcomes of LIT. The value of the interaction at each level of a factor indicates how it deviates from the reference level. The interaction between BA and age shows the value of BA2: Age2 is the largest, indicating patients between 19 and 27 years old (Age2) experienced more live births irrespective of BA conversion (Fig. 3E). Additionally, it is noteworthy that paternal blood type B is associated with increased live births, irrespective of BA conversion (Fig. 3F). This suggests that the paternal blood type may have a positive influence on the success of LIT in improving birth rates. Lastly, patients who tested positive for ACAIgM exhibit a reduced risk of miscarriages after undergoing LIT (Fig. 3G). This finding implies that the presence of ACAIgM antibodies may serve as a favourable indicator for the effectiveness of LIT in preventing miscarriages.

Overall, the empirical model and interaction analysis provide valuable insights into the effects of LIT and its interactions with age, paternal blood type, and ACAIgM in relation to live birth rates, contributing to a better understanding of the factors influencing successful pregnancy outcomes in RSA patients.

## Discussion

**Figure 4.**
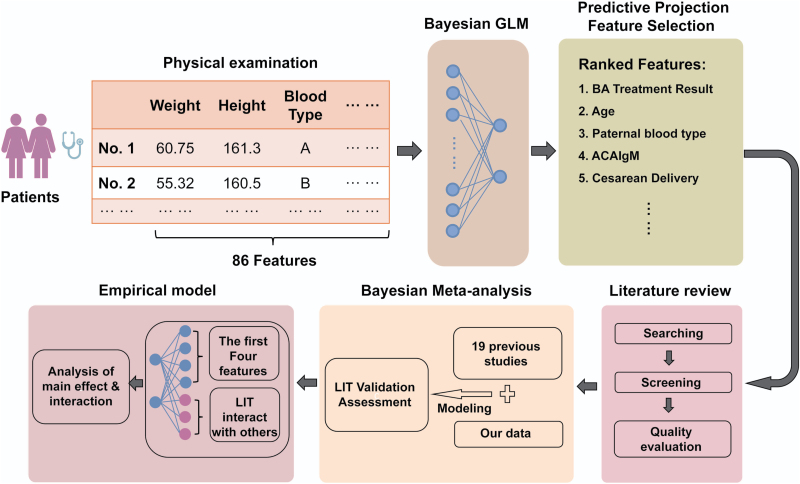
**Bayesian analysis pipeline for validating the efficacy of LIT.** The complete workflow consists of three steps: using Bayesian GLM to identify the signification of LIT in curing RSA, applying Bayesian meta-analysis to validate the conclusion of step 1, leveraging empirical model to identify potential candidates for LIT.

We have established a comprehensive Bayesian analysis workflow for evaluating the therapeutic efficacy of lymphocyte immunotherapy on RSA. The complete workflow is illustrated in Fig. 4. Our study provides compelling evidence supporting the significant influence of LIT on predicting parturition rates in RSA patients. This finding motivated us to conduct a Bayesian meta-analysis, which further confirmed the potential of LIT as a treatment for RSA. This is an encouraging outcome that emphasizes the need for clinical physicians to explore the utilization of LIT while being mindful of associated risks.

Moreover, to assist clinicians in selecting suitable candidates for LIT, we developed a comprehensive empirical model that considered factors such as model complexity, predictive performance, and interpretability. Through this model, we identified that the effectiveness of LIT varies depending on individual patient conditions. Specifically, LIT demonstrated higher efficacy in younger female patients whose male partners have blood type B and test positive for ACAIgM. Therefore, we recommend that patients meeting these criteria are the most suitable candidates for LIT treatment. Additionally, we advise patients at risk of RSA to consider starting their pregnancies only after testing positive for BAs, as this may help reduce the risk of miscarriages.

## Research limitations

There are still some limitations and drawbacks in our study. Firstly, we did not consider the interactions between LIT outcomes and the other 85 factors in the Bayesian GLM. This decision was made due to computational challenges associated with assessing high-order interactions and our primary focus on determining the effectiveness of LIT for RSA. Secondly, our criteria for selecting factors to establish the empirical model were based on a comprehensive consideration of interpretability and practicability. Nevertheless, there may be additional selection criteria that require further investigation and consideration of clinical conditions.

Additionally, our Bayesian meta-analysis combines data from retrospective studies with data from previous randomized trials to analyse the effectiveness of LIT for RSA. We adopted this approach because there is currently conflicting evidence regarding the efficacy of LIT. Introducing evidence from non-randomized studies into randomized trial data may result in transforming imprecise but unbiased estimation into precise but biased estimation, thereby converting uncertainty into error [35]. However, it is inevitable that this also introduces some degree of selection bias.

Furthermore, while our study demonstrated the effectiveness of LIT for RSA patients, the decision to undergo LIT should be made in consultation with the patient, taking into account their individual wishes and physical condition. As highlighted in our Bayesian meta-analysis, treatment responses may exhibit heterogeneity across different populations, necessitating careful consideration and individualized treatment approaches.

## Methods

### Statistical analysis

R programming language (version 4.4.2) [36] and Review manager (version 5.4) [37] were used for all analyses. The Bayesian model and meta-analysis were established using the rstanarm package [38] and rstan [39], respectively. The projpred package [23] was used for PPFS and forward search method was used (for more details about the statistical software we used, see Supplementary Tables S1–S3). To easily conduct literature search and management for Bayesian meta-analysis, we employed the Review Manager software (version 5.4).

### Data collection and processing

This study was conducted in collaboration with the Shenzhen Maternity and Child Healthcare Hospital, which is located at HongLi Road in Shenzhen, China. The data used for the Bayesian GLM analysis was collected retrospectively from clinic visits spanning the period between 2008 and 2020. The focus of the study was on patients with RSA, and the dataset included various types of information related to pregnancy outcomes, treatment records, paternal medical history, and other relevant diagnostic results. The dataset covered a wide range of factors, including parturition rate, medical history, immune indicators, infection inspection, endocrine level coagulation markers, chromosome examination, and male detection. Each examination consisted of multiple physical indicators that were associated with the patients. Samples with abnormal detection results were excluded from the analysis to ensure data quality and reliability. In order to assess the impact of each detection result on childbirth, we categorized the values of the detection results into different levels using a method that has practical significance. As an example, for the LIT variable, we divided it into three levels: not receiving LIT, receiving LIT with a negative BAs test, and receiving LIT with a positive BAs test. A total of 2316 patients were included in our analyses finally, and the criteria for patient inclusion and exclusion can be found in Supplementary Table S7. These criteria were established to ensure that the study population met the specific requirements and characteristics necessary for the research objectives.

To perform the Bayesian meta-analysis, we combined our data with previous published studies found via Google Scholar, Cochrane Library, and PubMed by using the subject terms ‘recurrent spontaneous abortion’ OR ‘recurrent miscarriage’ AND ‘blocking antibody’ OR ‘lymphocyte immunotherapy’. We also searched the Chinese literature from CNKI. Two authors extracted relevant data from each study independently into a Microsoft Excel spreadsheet, which included the country of affiliation of the first author, year of publication, trial design, inclusion criteria, study population, type of treatment, and treatment outcome in terms of live birth, however, to expand the data, we use successful pregnancy as the outcome if only pregnancy outcomes were reported in previous studies. Discrepancies in the sheet were investigated and resolved independently by a third author. Additionally, the Review manager was used to assess the quality of the included studies in the Bayesian meta-analysis (Supplementary Fig. S3).

For the inclusion criteria: only original articles reporting randomized controlled trials investigating the efficacy of LIT in the treatment of RSA patients were considered. Exclusion criteria: Studies were excluded if they were non-human studies, non-RCTs (non-randomized controlled trials), systematic reviews, or meta-analyses. A total of 1510 publications were found, from which 1491 were excluded for various reasons [40–58]. The quality of these studies was evaluated in Supplementary Fig.S3, all studies, except for [50]single blind, were randomized double-blind experiments, indicating the quality of these studies is high.

### Modelling the first model and statistical analysis

We first fitted a Bayesian multiple GLM using the stan programming language. For prior, we used relevant literatures to provide us with some prior knowledge (for details, see Supplementary Table S6) [9, 28–30, 59]. To account for factors where prior information was not available, we employed a flattened prior normal(0,1) for each level. For the prior on the intercept, we utilized the default prior of the stan_glm function. We used Markov chain Monte Carlo algorithm [17] (default option) for sampling, which takes a random walk through the parameter space, tending to walk in the high probability area and occasionally stepping into the low probability area to create a representation of the probability distribution on the parameters. Four chains were run in parallel for a total of 4000 iterations each with 2000 as warmup. The predictive projection feature was performed using the package projpred via forward search [32]. Therefore, in stan package our model was established as:


$$\begin{array}{l} ln\left(\frac{p(y=1|X)}{1-p(y=1|X))}\right)∼\beta_{0}+\beta_{1}x_{1}+\beta_{2}x_{2}+\ldots +\beta_{86}x_{86} \end{array}$$

$$\begin{array}{l} X=(x_{1},\;x_{2},\;\ldots ,x_{86}), \end{array}$$

where  p(y=1|X) is the probability of y being predicted as a positive case by a given X, in other words, it is the predicted probability of a patient successfully giving birth to a child by a given $X=(x_{1},\;x_{2},\ldots ,x_{86})$ and $x_{i}$ (i=1,2,3,…,86) represents various factors that might cause RSA.

### Bayesian meta-analysis

In our Bayesian meta-analysis, we combined our data with that of 13 previous studies to obtain a comprehensive analysis of the topic [60]. Bayesian methods provide a framework for conducting meta-analysis by treating both the data and model parameters as random quantities [61].

We established a Bayesian hierarchical model in which the observed effect size, represented by $Y_{J}$, as assumed to follow a normal distribution with mean $\theta_{J}$ and variance $\sigma_{J}^{2}$. The parameter $\theta_{J}$ represents the true effect size within each study, and it follows a normal distribution with mean μ and variance $\tau^{2}$.


$$\begin{array}{l} Y_{J}∼N\;(\theta_{J},\sigma_{J}^{2}) \end{array}$$

$$\begin{array}{l} \theta_{J}∼N\;(\mu ,\tau^{2}), \end{array}$$

To simplify the computational process, we transformed the model by expressing $\theta_{J}$ as a linear combination of μ and τ multiplied by η, where η is a standard normal random variable η∼N   (0,1). This transformation allows for faster computation of the model using the Stan code.


$$\begin{array}{l} \theta_{J}∼\mu +\tau *\eta \end{array}$$

The priors for μ and $\tau^{2}$ were specified as uniform distributions (stan default, see Supplementary Table S5). This allows the data to inform the posterior distribution and guide the estimation of the model parameters.

By applying this Bayesian hierarchical model, we were able to obtain probabilistic statements about the quantities of interest, such as the overall mean effect size and the between-study variance. This approach allows for a more comprehensive and nuanced analysis, incorporating uncertainty and accounting for heterogeneity among the included studies.

### Empirical model

From the PPFS, we chose LIT outcome, age, blood type, and ACAIgM as the features for the empirical model, which had an expected log pointwise predictive density of −1378.8 and an accuracy of 0.69. For prior, we set as Bayesian GLM (for details, see Supplementary Table S6). In stan package, we established the GLM as:


$$\begin{array}{l} ln\left(\frac{p(y=1|X)}{1-p(y=1|X))}\right)∼\beta_{0}+\underset{i=1}{\overset{4}{\sum }}\;\beta_{i}x_{i}+\underset{j=2}{\overset{4}{\sum }}\;\alpha_{j}(x_{1}\times x_{j}) \end{array}$$

$$\begin{array}{l} X=(x_{1},x_{2},x_{3},x_{4}), \end{array}$$

where  p(y=1|X) represents the predicted probability of a patient successfully giving birth to a child by a given $X=(x_{1},x_{2},x_{3},x_{4})$, and $x_{m}$ (m=1,2,3,4) represents the four features of empirical model, and “×”_’_ represents interaction between $x_{1}$ and other features.

### Research ethics

All participants in this study have been fully informed, and consent has been obtained. To ensure patient confidentiality, all identifiers that might reveal a patient’s identity have been removed from the manuscript.

## Data availability

The data and code that support the findings of this study are openly available in Bayesian-Interpretation-of-RSA-Treatment at github.com/10406502403/Bayesian-Interpretation-of-RSA-Treatment.

## Supplementary Material

lnad049_suppl_Supplementary_Tables_S1-S9_Figures_S1-S3

lnad049_suppl_Supplementary_Materials_S1

lnad049_suppl_Supplementary_Materials_S2

lnad049_suppl_Supplementary_Materials_S3

lnad049_suppl_Supplementary_Materials_S4

## References

[1] Dimitriadis E, Menkhorst E, Saito S, et al. Recurrent pregnancy loss. Nat Rev Dis Primers 2020;6:1–19. 33303732 10.1038/s41572-020-00228-z

[2] Rai R, Regan L. Recurrent miscarriage. Lancet 2006;368:601–11. 16905025 10.1016/S0140-6736(06)69204-0

[3] Practice Committee of the American Society for Reproductive Medicine. Evaluation and treatment of recurrent pregnancy loss: a committee opinion. Fertil Steril 2012;98:1103–11. 22835448 10.1016/j.fertnstert.2012.06.048

[4] Sacha, Krieg, Lynn, Westphal. Immune function and recurrent pregnancy loss. Semin Reprod Med 2015;33:305–12. 26132935 10.1055/s-0035-1554917

[5] Howard Carp. Immunotherapy for recurrent pregnancy loss. Best Pract Res Clin Obstet Gynaecol 2019;60:77–86. 31521575 10.1016/j.bpobgyn.2019.07.005

[6] Mowbray JF, Liddell H, Underwood JL, et al. Controlled trial of treatment of recurrent spontaneous abortion by immunisation with paternal cells. Lancet 1985;325:941–3. 10.1016/s0140-6736(85)91723-4 2859409

[7] Coulam CB, Clark DA, Collins J, et al. Worldwide collaborative observational study and meta-analysis on allogenic leukocyte immunotherapy for recurrent spontaneous abortion. Am J Reprod Immunol 2013;32:55–72. 7826502

[8] Francisco PD, Tan-Lim CSC, Agcaoili-De Jesus MSL. Efficacy of lymphocyte immunotherapy in the treatment of recurrent pregnancy loss from alloimmunity: a systematic review and meta-analysis. Am J Reprod Immunol 2022;88:e13605. 35894648 10.1111/aji.13605

[9] Liu Z, Xu H, Kang X, et al. Allogenic lymphocyte immunotherapy for unexplained recurrent spontaneous abortion: a meta-analysis. Am J Reproduct Immunol 2016;76:443–53. 10.1111/aji.12511 27105633

[10] Pan X, Jiang J, Ma Q, et al. Outbreak of HIV infection linked to nosocomial transmission, China, 2016–2017. Emerg Infect Dis 2018;24:2141. 30457542 10.3201/eid2412.180117 PMC6256388

[11] Ticconi C, Pietropolli A, Simone ND, et al. Endometrial immune dysfunction in recurrent pregnancy loss. Int J Mol Sci 2019;20:5332. 31717776 10.3390/ijms20215332 PMC6862690

[12] Ford HB, Schust DJ. Recurrent pregnancy loss: etiology, diagnosis, and therapy. Rev Obstet Gynecol 2009;2:76–83. 19609401 PMC2709325

[13] Carp H. Immunotherapy for recurrent pregnancy loss. Best Pract Res Clin Obstetr Gynaecol 2019;60:77–86. 10.1016/j.bpobgyn.2019.07.005 31521575

[14] Dey DK, Ghosh SK, Mallick BK. Generalized Linear Models: A Bayesian Perspective. CRC Press, 2000.

[15] Faraway JJ. Extending the Linear Model with R: Generalized Linear, Mixed Effects and Nonparametric Regression Models, 2006.

[16] West BT. Bayesian analysis of between-group differences in variance components in hierarchical generalized linear models. JSM Proceedings, Survey Research Methods Section. Alexandria, VA: American Statistical Association, 1828, 1842.

[17] Kruschke JK. Bayesian data analysis. Wiley Interdiscip Rev Cognit Sci 2010;1:658–76. 26271651 10.1002/wcs.72

[18] Kruschke, J. Doing Bayesian Data Analysis: A Tutorial with R, JAGS, and Stan, ELSEVIER, 2014.

[19] Kruschke JK. Bayesian analysis reporting guidelines. Nat Hum Behav 2021;5:1282–91. 34400814 10.1038/s41562-021-01177-7 PMC8526359

[20] Brooks S, Gelman A, Jones G, Meng X-L. Handbook of Markov Chain Monte Carlo. CRC Press, 2011.

[21] Gelman A, Carlin JB, Stern HS, Rubin DB. Bayesian Data Analysis. Chapman and Hall/CRC, 1995.

[22] Hastings WK. Monte Carlo sampling methods using Markov chains and their applications. Biometrika, 1970;57:97–109.

[23] Piironen J, Paasiniemi M, Vehtari A. Projective inference in high-dimensional problems: prediction and feature selection. Electron J Stat 2020;14:2155–97.

[24] Vehtari A, Gelman A, Gabry J. Practical Bayesian model evaluation using leave-one-out cross-validation and WAIC. Stat Comput 2017;27:1413–32.

[25] Williams DR, Rast P, Bürkner P-C. Bayesian meta-analysis with weakly informative prior distributions, *PsyArXiv* 2018, 10.31234/osf.io/7tbrm.

[26] Cline AM, Kutteh WH. Is there a role of autoimmunity in implantation failure after in-vitro fertilization? Curr Opin Obstet Gynecol 2009;21:291–5. 19469047 10.1097/gco.0b013e3283294879

[27] Hořejší J, Martinek J, Nováková D, et al. Autoimmune antiovarian antibodies and their impact on the success of an IVF/ET program. Ann N Y Acad Sci 2000;900:351–6. 10818424 10.1111/j.1749-6632.2000.tb06248.x

[28] Magnus MC, Wilcox AJ, Morken N-H, et al. Role of maternal age and pregnancy history in risk of miscarriage: prospective register based study. Bmj 2019;364:l869. 30894356 10.1136/bmj.l869 PMC6425455

[29] Cavalcante MB, Cavalcante CTMB, Sarno M, et al. Antinuclear antibodies and recurrent miscarriage: systematic review and meta-analysis. Am J Reprod Immunol 2020;83:e13215. 31821640 10.1111/aji.13215

[30] Li Y, Li X, Luo S. Effect of Recurrent Spontaneous Abortion on Pregnancy Outcomes in Sequent Successful Pregnancy Patients. Journal of Guangzhou University of Traditional Chinese Medicine 2015, 979–83.

[31] Ruiz-Irastorza G, Crowther M, Branch W, et al. Antiphospholipid syndrome. Lancet 2010;376:1498–509. 20822807 10.1016/S0140-6736(10)60709-X

[32] Vinatier D, Dufour P, Cosson M, et al. Antiphospholipid syndrome and recurrent miscarriages. Eur J Obstetr Gynecol Reproduct Biol 2001;96:37–50. 10.1016/s0301-2115(00)00404-8 11311759

[33] Abdullahi ZG, Abdul MA, Aminu SM, et al. Antiphospholipid antibodies among pregnant women with recurrent fetal wastage in a tertiary hospital in Northern Nigeria. Ann Afr Med 2016;15:133–7. 27549418 10.4103/1596-3519.188894 PMC5402808

[34] Kiernan D. Natural resources biometrics (online book). 2021; Chapter 6.1, stats.libretexts.org/@go/page/2904.

[35] Higgins JP, Green S. Cochrane handbook for systematic reviews of interventions,Wiley Online Library 2008.

[36] Team, R. C. R: A language and environment for statistical computing. MSOR Connections 2014;1:275–86.

[37] Deeks JJ, Higgins JP. Statistical algorithms in review manager 5. Statistical Methods Group of The Cochrane Collaboration 2010;1:1–11.

[38] Muth C, Oravecz Z, Gabry J. User-friendly Bayesian regression modeling: a tutorial with rstanarm and shinystan. Quant Methods Psychol 2018;14:99–119.

[39] Jewson, J. RStan: Efficient MCMC in R. 2017, dokumen.tips/documents/rstan-efficient-mcmc-in-r-university-of-warwick-generated-quantities-optional.html?page=20

[40] Cui Y, Zhong X, Ban Q, et al. Study of immunotherapy with lymphocytes in women with recurrent spontaneous abortion. Modern Prev Med 2011;38:1626–7.

[41] Hong L, Huaixiu W, Jing W. The lymphocyte injects and treats the habitual abortion that the immune factor causes. Med Mag Shanxi 2003;32:308–9.

[42] Mowbray J, Liddell H, Underwood JL, et al. Controlled trial of treatment of recurrent spontaneous abortion by immunisation with paternal cells. Lancet 1985;325:941–3. 10.1016/s0140-6736(85)91723-4 2859409

[43] Lin S, Yan S, Shan E. Analysis the efficacy of immunotherapy with lymphocytes for recurrent spontaneous abortion. Jilin Med 2012;33:1822–3.

[44] Pandey MK, Agrawal S. Induction of MLR-Bf and protection of fetal loss: a current double blind randomized trial of paternal lymphocyte immunization for women with recurrent spontaneous abortion. Int Immunopharmacol 2004;4:289–98. 14996420 10.1016/j.intimp.2004.01.001

[45] Chen J-L, Yang J-M, Huang Y-Z, et al. Clinical observation of lymphocyte active immunotherapy in 380 patients with unexplained recurrent spontaneous abortion. Int Immunopharmacol 2016;40:347–50. 27673476 10.1016/j.intimp.2016.09.018

[46] Liu S, Gu X, Weng R. Clinical effect of lymphocyte immunotherapy on patients with unexplained recurrent spontaneous abortion. Immun Inflammation Dis 2021;9:1272–8. 10.1002/iid3.474 PMC8589387 34102013

[47] Carp H et al. Allogenic leukocyte immunization after five or more miscarriages Recurrent Miscarriage Immunotherapy Trialists Group. Hum Reprod 1997;12:250–5. 9070705 10.1093/humrep/12.2.250

[48] Illeni MT, Marelli G, Parazzini F, et al. Immunology: immunotherapy and recurrent abortion: a randomized clinical trial. Hum Reprod 1994;9:1247–9. 7962426 10.1093/oxfordjournals.humrep.a138687

[49] Sarno M, Cavalcante MB, Niag M, et al. Gestational and perinatal outcomes in recurrent miscarriages couples treated with lymphocyte immunotherapy. Eur J Obstetr Gynecol Reproduct Biol 2019;3:100036. 10.1016/j.eurox.2019.100036 PMC6687386 31403124

[50] Aiwu W, Mingzhu L, Runzhi W. Preventive treatment of unexplained recurrent spontaneous abortion and effect on pregnancy outcome by lymphocytes immunotherapy. China J Chin Med 2013;28:876–8.

[51] Bin T, Kaishu H, Wenquan Z. Analysis of lymphocytes immunotherapy for recurrent spontaneous abortion. Chin Med Treat Works 2013;21:171–2.

[52] Ho HN, Gill TJ, Hsieh HJ, et al. Immunotherapy for recurrent spontaneous abortions in a Chinese population. Am J Reproduct Immunol 1991;25:10–5. 10.1111/j.1600-0897.1991.tb01056.x 2029326

[53] Group RMIT. Worldwide collaborative observational study and meta-analysis on allogenic leukocyte immunotherapy for recurrent spontaneous abortion 1. Am J Reprod Immunol 1994;32:55–72. 7826502

[54] Gatenby PA, Cameron K, Simes RJ, et al. Treatment of recurrent spontaneous abortion by immunization with paternal lymphocytes: results of a controlled trial. Am J Reproduct Immunol 1993;29:88–94. 10.1111/j.1600-0897.1993.tb00571.x 8329110

[55] Daya S, Gunby J; Group RMIT. The effectiveness of allogeneic leukocyte immunization in unexplained primary recurrent spontaneous abortion. Am J Reprod Immunol 1994;32:294–302. 7718097 10.1111/j.1600-0897.1994.tb01129.x

[56] Clark DA, Daya S. Trials and tribulation in the treatment of recurrent spontaneous abortion. Am J Reproduct Immunol 1991;25:18–24. 10.1111/j.1600-0897.1991.tb01058.x 2029328

[57] Cauchi M, Lim D, Young D, et al. Treatment of recurrent aborters by immunization with paternal cells—controlled trial. Am J Reprod Immunol 1991;25:16–7. 2029327 10.1111/j.1600-0897.1991.tb01057.x

[58] Christiansen OB, Mathiesen O, Husth M, et al. Placebo-controlled trial of active immunization with third party leukocytes in recurrent miscarriage. Acta Obstet Gynecol Scand 1994;73:261–8. 8122510 10.3109/00016349409023451

[59] Bandyopadhyay AR, Chatterjee D, Chatterjee M, et al. Maternal fetal interaction in the ABO system: a comparative analysis of healthy mother and couples with spontaneous abortion in Bengalee population. Am J Human Biol 2011;23:76–9. 21080445 10.1002/ajhb.21102

[60] Guzzo RA, Jackson SE, Katzell RA. Meta-analysis analysis. Res Organ Behav 1987;9:407–42.

[61] Sutton AJ, Abrams KR. Bayesian methods in meta-analysis and evidence synthesis. Stat Methods Med Res 2001;10:277–303. 11491414 10.1177/096228020101000404

