# Supplementary material for: A comprehensive Bayesian analysis assessing the effectiveness of lymphocyte immunotherapy for recurrent spontaneous abortion
Source: Life Med. 2023 Dec 8;2(6):lnad049. doi: 10.1093/lifemedi/lnad049 (PMC11749857; doi:10.1093/lifemedi/lnad049)
Supplement: lnad049_suppl_Supplementary_Tables_S1-S9_Figures_S1-S3 [file lnad049_suppl_Supplementary_Tables_S1-S9_Figures_S1-S3.pdf]

R package and version

1. Bayesian GLM:

Table S1: R package and version of Bayesian GLM

R package	version
projpred	2.2.1
rstanarm	2.21.3
tidyr	1.2.1
dplyr	1.0.10
ggplot2	3.3.6
bayesplot	1.9.0
forestplot	3.1.0
sjPlot	2.8.11
sjlabelled	1.2.0
sjmisc	2.8.9
readxl	1.4.1
gridExtra	2.3.0

2. Bayesian meta-analysis:

Table S2: R package and version of Bayesian meta-analysis

R package	version
rstan	2.21.7
bayesplot	1.9.0
ggplot2	3.3.6

3. Empirical model:

Table S3: R package and version of empirical model

R package	version
projpred	2.2.1
rstanarm	2.21.3
tidyr	1.2.1
dplyr	1.0.10
ggplot2	3.3.6
bayesplot	1.9.0
forestplot	3.1.0
sjPlot	2.8.11
gridExtra	2.3.0
sjlabelled	1.2.0
sjmisc	2.8.9
readxl	1.4.1

Prior

The detailed of priors are shown as follows:

1. Bayesian GLM

Table S4: The prior distributions of Parameters of Bayesian GLM

Parameters	Prior distribution
BA2	Normal (1.4104, 1)
BA3	Normal (1.4104, 1)
Age2	Normal (-0.2543, 1)
Age3	Normal (0.3129, 1)
Age4	Normal (0.4416, 1)
Age5	Normal (0.4416, 1)
Age6	Normal (-0.3026, 1)
BT2	Normal (-0.2445, 1)
BT3	Normal (-1.9627, 1)
BT4	Normal (0.1217, 1)
BT5	Normal (0.3131, 1)
ANA2	Normal (-1.1248, 1)
ANA3	Normal (0.3131, 1)
ACA2	Normal (0.1614, 1)
ACA3	Normal (0.1149, 1)
Others	Normal (0, 1)

2. Bayesian meta-analysis

Table S5: The prior distributions of Parameters of Bayesian meta-analysis

Parameters	Prior distribution
μ	Uniform distribution
τ	Uniform distribution

3. Empirical model

Table S6: The prior distributions of Parameters of empirical model

Parameters	Prior distribution
BA2	Normal (1.4104, 1)
BA3	Normal (1.4104, 1)
Age2	Normal (-0.2543, 1)
Age3	Normal (0.3129, 1)
Age4	Normal (0.4416, 1)
Age5	Normal (0.4416, 1)
Age6	Normal (-0.3026, 1)
BT2	Normal (-0.2445, 1)
BT3	Normal (-1.9627, 1)
BT4	Normal (0.1217, 1)
BT5	Normal (0.3131, 1)
ACA2	Normal (-0.4431, 1)
ACA3	Normal (0.1149, 1)
Others	Normal (0, 1)

Inclusion and exclusion criteria of our data

Table S7: Inclusion and exclusion criteria

Inclusion Criteria	Exclusion Criteria
Patients must be 18 years old or above	Patients were less than 18 years old
Patients did not suffer from any severe mental illness or poor general health during the treatment.	Patients suffered from any severe mental illness or poor general health during the treatment.
Patients suffered from spontaneous miscarriages at least three times	Patients suffered from spontaneous miscarriages less than three times
Patients agreed to record their medical history, test results and therapeutic outcome in the file and accepted follow-up interviews	Patients were not willing to record their medical history, test results and therapeutic outcome in the file and participate in follow-up interviews.
Patients had at least two follow-up visits	Patients had less than two follow-up visits
Patients had at least three items of inspection	Patients had less than three items of inspection
Patients had at least three courses of LIT or two courses of traditional treatment	Patients had less than three courses of LIT or two courses of traditional treatment
Patients had a comprehensive information of donor who had all inspections to guarantee the safety of LIT.	Patients had an incomplete information of donor who did not have enough inspections so that the doctor cannot guarantee the safety of LIT.

Table S8. Trails included in this article

Author	Treatment			Control		Random effect	Relative risk
	year	d1	n1	d0	n0	(95% CI)	(95%CI)
Mowbray JF	1985	25	37	14	30	1.51 [1.01,2.23]	1.45[0.93,2.25]
Cauchi MN	1991	13	20	16	22	1.02[0.70,1.49]	0.89[0.59,1.35]
HO HONG - NERNG	1991	33	42	32	49	1.24[0.96,1.59]	1.20[0.93,1.56]
CLARK DAVID A	1991	7	11	2	7	1.84[0.90,3.80]	2.23[0.64,7.80]
GATENBY PAUL A	1993	13	19	12	20	1.26[0.82,1.93]	1.14[0.71,1.83]
Coulam CB	1994	6	10	5	12	1.58[0.85,2.84]	1.44[0.62,3.33]
Reznikoff-ET MF	1994	17	26	14	26	1.32[0.88,1.95]	1.21[0.77,1.91]
Illeni Maria Teresa	1994	10	16	11	14	0.96[0.63,1.46]	0.80[0.50,1.27]
Christiansen Ole B	1994	31	48	16	28	1.22[0.85,1.72]	1.13[0.77,1.66]
Carp HJ	1997	5	11	11	31	1.47[0.79,2.67]	1.28[0.57,2.86]
Hong L	2003	18	21	2	8	2.11[1.06,4.42]	3.43[1.02,11.53]
Pandey Manoj Kumar	2004	21	25	6	20	2.25[1.36,3.90]	2.80[1.40,5.59]
Cui YP	2011	41	49	24	45	1.59[1.19,2.10]	1.57[1.16,2.12]
Lin S	2012	33	42	17	42	1.89[1.31,2.69]	1.94[1.30,2.89]
Aiwu W	2013	250	297	254	591	1.95[1.76,2.17]	1.96[1.76,2.18]
Bin T	2013	32	39	18	39	1.76[1.27,2.46]	1.78[1.23,2.57]
Chen Jian-Ling	2016	341	380	119	369	2.73[2.35,3.18]	2.78[2.39,3.24]
Sarno	2019	452	752	114	344	1.81[1.54,2.12]	1.81[1.54,2.13]
Sudong Liu	2021	233	444	39	260	3.19[2.38,4.52]	3.50[2.59,4.73]
This article	2022	1370	2109	36	207	3.38[2.53,4.60]	3.74[2.77,5.03]
Total		2951	4398	762	2164	1.69[0.69,4.13]	

Table S9. The accuracy and expected log predictive density of the Bayesian model determined by 19 features based on predictive projection feature selection

Number	Feature
1	BA Treatment Result
2	Age
3	Blood Type
4	Anticardiolipin Antibody IgM
5	Cesarean Delivery Times
6	Menstrual Cycle Day
7	FT3
8	Spotaneous Abortion Times
9	Antithrombin III AT III
10	AMH
11	T
12	Non operation History
13	BMI
14	NK Cell
15	Rountine B
16	Blocking Antibody
17	Protein S
18	Homocysteine
19	Antisperm Antibody

Models Check:

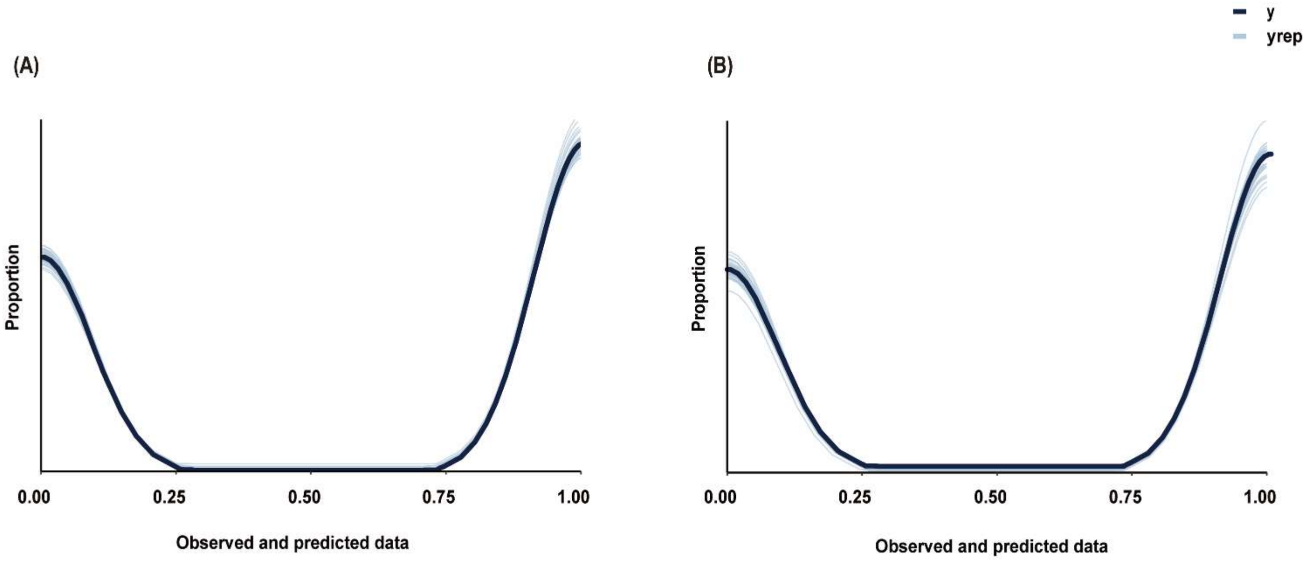

Figure S1. Posterior predictive check.

(A) Bayesian multiple regression model. (B) Empirical model. The black (y) and blue (y_{rep}) curves represent the observed and predicted data, respectively. The horizontal axis represents the values of y (observed or predicted), and the vertical axis represents the proportion of the total population that corresponds to each value.

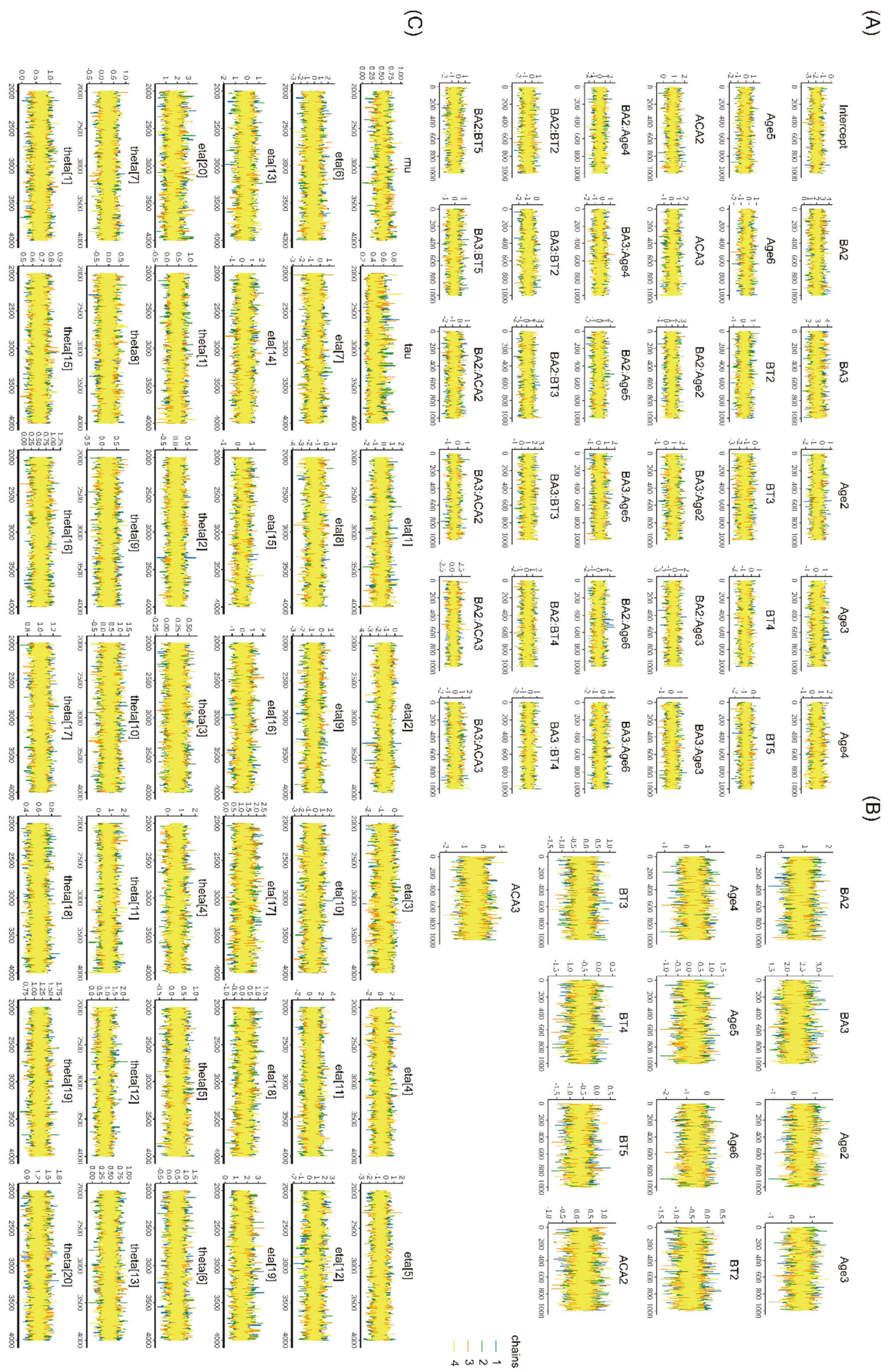

Figure S2. Trace plots showing the iteration number against the parameter value for the factors of our models.

(A) Convergence of Bayesian GLM. Here, we only show the convergence of the top 4 factors. The convergence of other factors follows a similar trend. (B) Convergence of empirical model. The four MCMC chains converge

to the same region without any obvious dispersion in these two models, indicating that they are effective. (C) Convergence of Bayesian meta-analysis.BA2 and BA3 refer to patients with or without BA conversion, respectively. Age2-Age6 refer to patients aged between 19-27, 27-29, 29-31, 31-34, and 34-57 years old. BT2-BT5 refers to the paternal blood types A, AB, B, or O. ACA2-ACA3 refers to patients with anticardiolipin antibody IgM tested negative or positive, respectively.

Bayesian Meta-analysis

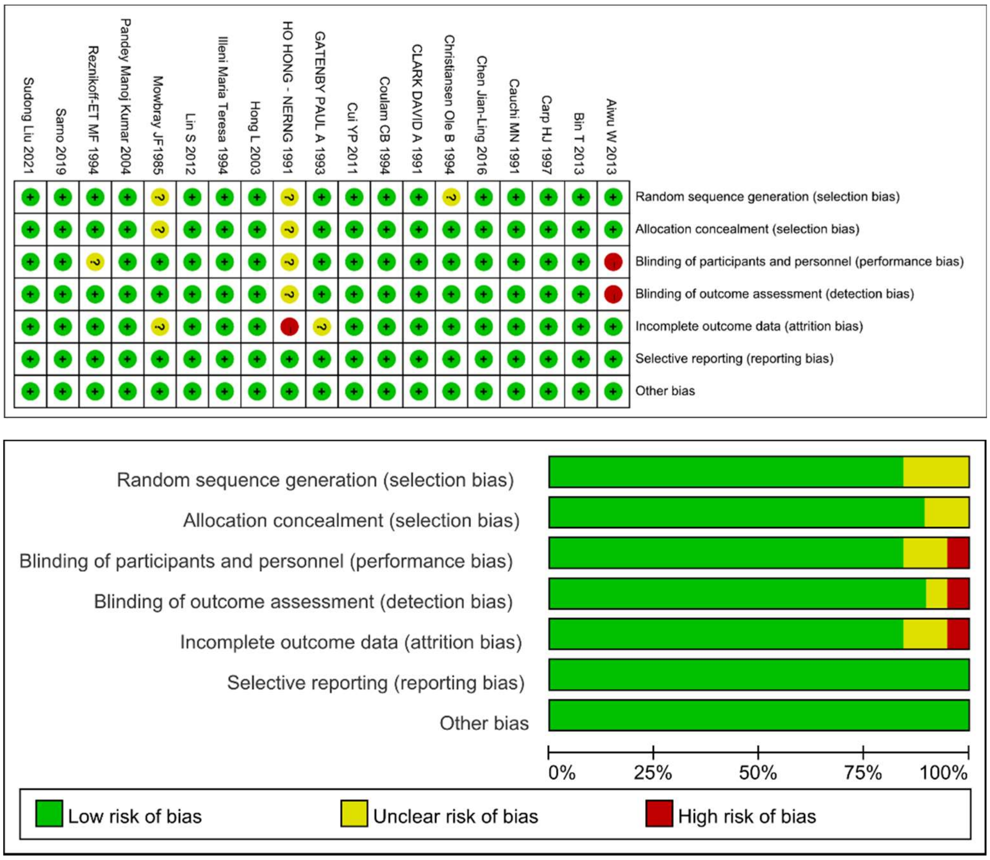

Figure S3. Quality assessment of included studies.
